# Distinct Stress Appraisal Dimensions, Insomnia, and Physical Activity as Differential Pathways to Psychological Distress: A Cross-Sectional Study in Tunisian University Students

**DOI:** 10.3390/healthcare14142056

**Published:** 2026-07-09

**Authors:** Taoufik Selmi, Souhail Bchini, Jaouher Hamaidi, Ahmed M. Abdelsalam, Halil İbrahim Ceylan, Raul Ioan Muntean, Lolwa Barakat, Noureddine M. Ben Said, Ismail Dergaa, Noomen Guelmami, Nasr Chalghaf, Fairouz Azaiez

**Affiliations:** 1 High Institute of Sport and Physical Education of Ksar Said, University of Manouba, Manouba 2010, Tunisia; taoufikselmi72@gmail.com (T.S.);; 2 Department of Human and Social Sciences, High Institute of Sport and Physical Education of Kef, University of Jendouba, Kef 7100, Tunisia; souhail.bchini@gmail.com (S.B.); hamaidijaouher@gmail.com (J.H.);; 3 Department of Biomechanics and Motor Behavior, College of Sport Sciences and Physical Activity, King Saud University, Riyadh 12371, Saudi Arabia; amohmed@ksu.edu.sa (A.M.A.);; 4 Department of Physical Education and Sports, Faculty of Sport Sciences, Ataturk University, Erzurum 25240, Turkey; 5 Department of Physical Education and Sport, Faculty of Law and Social Sciences, University “1 Decembrie 1918” of Alba Iulia, 510009 Alba Iulia, Romania; 6 Clinical Research Center, USC Institute for Addiction Science, Keck School of Medicine, University of Southern California, Los Angeles, CA 90089, USA; 7 Research Unit Physical Activity, Sport, and Health, UR18JS01, National Observatory of Sport, Tunis 1003, Tunisia; 8 Department of Human Sciences, Higher Institute of Sport and Physical Education of Gafsa, University of Gafsa, Gafsa 2112, Tunisia

**Keywords:** anxiety, coping strategies, DASS-21, helplessness, insomnia, mediation analysis, perceived stress, physical activity, PSS-10, sleep, structural equation modeling, young adults

## Abstract

**Background:** Young adults face a convergence of academic, economic, and social pressures that elevates their risk for stress-related psychological disorders. Perceived stress encompasses two theoretically and empirically distinct components, perceived helplessness and perceived self-efficacy, but most research treats it as a single composite score, obscuring the differential mechanisms through which each dimension influences mental health. **Aim:** The study aimed to (i) quantify the direct effects of perceived helplessness and perceived self-efficacy on psychological distress, (ii) test insomnia and physical activity as concurrent mediators of each dimension’s indirect pathway to distress, and (iii) determine whether the two stress dimensions activate meaningfully distinct mechanisms. **Methods:** A cross-sectional study enrolled 1688 young adults aged 18–23 years (M = 18.76, SD = 1.01; 41.6% female) from higher education institutions in Tunisia. Participants completed Arabic-validated versions of the Perceived Stress Scale (PSS-10), Insomnia Severity Index (ISI), Depression Anxiety Stress Scales (DASS-21), and the International Physical Activity Questionnaire (IPAQ). Structural equation modeling (SEM) with unweighted least squares estimation and 5000 bootstrap resamples tested direct and indirect pathways. **Results:** Perceived helplessness exerted strong direct effects on psychological distress (beta = 0.25, *p* < 0.001) and substantial indirect effects through insomnia (beta = 0.21, *p* < 0.001) and physical activity (beta = 0.03, *p* < 0.001). Insomnia was the dominant mediator, showing the strongest bivariate association with distress (r = 0.58, *p* < 0.01). Perceived self-efficacy produced markedly weaker direct (beta = 0.06, *p* = 0.002) and indirect effects, a pattern that, like its other associations in the model, runs in the same positive direction as perceived helplessness rather than the inverse direction conventionally expected of a protective factor. Model fit was acceptable (CFI = 0.994, SRMR = 0.018, RMSEA = 0.076, the latter below the conventional 0.08 threshold). **Conclusions:** Stress management deficits are associated with psychological distress primarily through sleep disruption, consistent with a mediating role for insomnia in this cross-sectional sample. Physical activity provides a meaningful but secondary protective correlate. These findings offer a preliminary, hypothesis-generating basis for stratified, mechanism-based mental health interventions targeting sleep regulation and stress management in young adult populations, pending prospective confirmation.

## 1. Introduction

Young adults occupy a developmental window of compounded psychological vulnerability. Academic demands, economic uncertainty, occupational transitions, and shifting social identities converge to produce stress-related disorders at rates exceeding those observed in older cohorts [1,2]. Globally, depression and anxiety disorders affect an estimated 15–20% of individuals aged 15–24 years, with college students consistently among the highest-risk subgroups [3,4]. The health burden of chronic stress is not confined to subjective distress: it disrupts sleep architecture, suppresses immune function, and accelerates cardiovascular and metabolic risk trajectories even in otherwise healthy individuals [5,6]. Critically, the transition to higher education exposes young people to concentrated stressors at a stage when behavioral habits, including sleep patterns and physical activity routines, are still being consolidated, making this population both uniquely susceptible and uniquely accessible for preventive intervention [5,6]. Stress is not a unitary experience. Lazarus and Folkman’s transactional model identifies two distinct appraisal processes that mediate the stress-health relationship [7]: primary appraisal, in which the individual evaluates whether a situation exceeds available capacities, and secondary appraisal, in which available coping resources are evaluated. These two processes are not equivalent dimensions of the same construct; they activate different regulatory responses, engage different behavioral consequences, and should operate through distinct pathways to psychological outcomes. Achieving precision in stress research and in the design of targeted interventions requires treating them as separate predictors rather than as inverse poles of a single score [8,9].

The Perceived Stress Scale (PSS-10), developed by Cohen and colleagues [10], provides two factorially distinct subscales operationalizing these appraisal dimensions. The negative subscale captures perceived helplessness, defined as the appraisal of insufficient personal control over external demands. The positive subscale captures perceived self-efficacy, reflecting confidence in one’s capacity to manage those demands. When treated as separate predictors, these subscales yield substantively different patterns of association with psychological outcomes [8,9]. Perceived helplessness is more consistently linked to emotional dysregulation, rumination, sleep disturbance, and clinical levels of anxiety and depression; perceived self-efficacy is more closely tied to proactive coping, health behavior maintenance, and resilience to acute stressors [11,12]. It should be noted, however, that this protective characterization is not universal: some prior work suggests that the association between perceived self-efficacy and distress-related outcomes can be inconsistent or context-dependent rather than uniformly inverse [11,13], and the present study is positioned, in part, to examine whether this protective pattern holds once perceived self-efficacy is modeled as empirically distinct from perceived helplessness rather than assumed a priori. Two behavioral and physiological pathways are particularly positioned to mediate these divergent relationships. Sleep disturbance, and insomnia in particular, occupies a central position in the stress-mental health interface. The neurobiological basis of this centrality lies in the role of non-REM slow-wave sleep in cortical restoration and REM sleep in emotional memory consolidation: when stress activates the hypothalamic–pituitary–adrenal axis and sustains arousal, sleep onset and maintenance are compromised, selectively impairing the regulatory processes needed to restore emotional equilibrium [14]. Insomnia is simultaneously a consequence of unmanaged stress and an amplifier of subsequent distress, creating a self-perpetuating cycle that is difficult to interrupt without addressing both sleep and stress together [15]. Physical activity occupies the complementary position in this model. Regular participation in moderate-to-vigorous activity is associated with attenuated physiological stress reactivity and with lower levels of depression and anxiety symptoms across adult populations [16,17]. Physical activity engagement requires motivational resources and behavioral self-regulation, capacities that diminish under conditions of high perceived helplessness; simultaneously, stress directly displaces discretionary activity through time pressure and fatigue [18,19]. These interdependencies suggest that insomnia and physical activity may function as concurrent mediating pathways through which the two stress appraisal dimensions differentially reach psychological distress, a hypothesis that has not yet been formally tested within a unified structural model.

Three specific gaps limit the current evidence base and motivate the present investigation. First, the large majority of studies on stress and mental health in young adults operationalize the PSS-10 as a total composite score, sacrificing the differential information contained in its two subscales [8,9,20]. This is consequential: if perceived helplessness and perceived self-efficacy operate through different mechanisms, pooling them conceals which pathway drives distress and which behavioral intervention would most efficiently interrupt it. Second, insomnia and physical activity have been examined as separate mediators of the stress-mental health relationship in independent research traditions [15,21], but rarely within a single model estimating both simultaneously. Examining them in isolation cannot resolve whether they represent complementary mechanisms producing additive effects or competing pathways with differential magnitudes across stress dimensions. Third, a targeted search of the literature did not identify a prior study integrating both PSS-10 subscales with insomnia and physical activity within a unified structural model in a large young-adult sample; the closest related studies examined these elements only in partial combination [22], leaving unanswered the fundamental clinical question: Does perceived helplessness exert its damage primarily through sleep disruption, while perceived self-efficacy exerts its protective influence primarily through physical activity engagement? The answer carries direct intervention implications, since sleep-focused treatment approaches and activity promotion programs occupy distinct resource streams in student health systems, and evidence on which stream to prioritize for whom currently lacks a structural foundation.

To address these gaps, the present study employed structural equation modeling in a large sample of 1688 Tunisian young adults enrolled in higher education institutions. Arabic-validated assessments of perceived helplessness and perceived self-efficacy (PSS-10 subscales), insomnia severity (ISI), physical activity levels (IPAQ), and psychological distress (DASS-21) were collected and tested within a comprehensive model of direct and indirect pathways. The study aimed to (i) quantify the direct effects of perceived helplessness and perceived self-efficacy on psychological distress, (ii) test insomnia and physical activity as concurrent mediators of each stress dimension’s indirect pathway to distress, and (iii) determine whether the two dimensions activate meaningfully distinct mechanisms. We hypothesized that perceived helplessness would exert its primary indirect effect through insomnia (H1), that perceived self-efficacy would exert its primary indirect effect through physical activity (H2), and that the insomnia pathway would substantially exceed the physical activity pathway for perceived helplessness but not for perceived self-efficacy (H3).

## 2. Materials and Methods

### 2.1. Ethical Approval

This study was conducted between September 2023 and February 2024 in accordance with the Declaration of Helsinki. The research protocol was reviewed and approved by the Ethics Committee of the High Institute of Sport and Physical Education of Kef, University of Jendouba (approval code: PHS-05a/2023; approved on 4 May 2023). Ethical oversight was supported by the University of Jendouba’s digital research-ethics management framework [23]. All procedures adhered to current international ethical guidelines for research practice in sports medicine and exercise science [24]. Written informed consent was obtained from all participants before data collection. No personally identifiable information was collected, and all data were analyzed at the aggregate level.

### 2.2. Sample Size

Sample size estimation was guided by structural equation modeling requirements and benchmarks from comparable studies. Manzar and colleagues [15] demonstrated medium-to-large effect sizes linking perceived stress to psychological distress through insomnia in 475 university students, while Chen and colleagues [11] documented comparable mediation magnitudes linking personality traits to psychological distress through stress mindset and coping flexibility in a 260-participant undergraduate sample. Based on these benchmarks and established sample-size guidance for structural equation modeling, which recommends a minimum of approximately 200 cases together with at least 10 to 20 participants per estimated parameter for stable estimation, the minimum required sample for the planned SEM was set at approximately 200 participants. The enrolled sample of 1688 substantially exceeds this requirement, providing robust estimation precision and high power for detecting even small indirect effects.

### 2.3. Participants

A total of 1688 young adults participated (males: n = 985, 58.4%; females: n = 703, 41.6%), with a mean age of 18.76 years (SD = 1.01, range: 18–23). Participants were recruited from higher education institutions across Tunisia, specifically six public institutions located across three regions of the country (Greater Tunis, the North-West, and the Center), reflecting the institutional affiliations of the contributing research teams over a six-month data collection period using a non-probability convenience sampling approach. The study targeted young adults, given their well-documented vulnerability to stress-related psychological difficulties during the academic transition period [1,5]. Data were collected via online standardized self-report instruments. Inclusion criteria were: (i) age between 18 and 23 years; (ii) current enrollment in a higher education institution; and (iii) ability to complete the survey independently in Arabic. Individuals with pre-existing diagnosed psychiatric conditions requiring ongoing pharmacological treatment were excluded, assessed via a single self-report screening item asking whether the participant had ever received a formal psychiatric diagnosis requiring ongoing pharmacological treatment, as such conditions could confound the relationship between perceived stress and psychological distress outcomes.

### 2.4. Study Design

This cross-sectional study used a structured online survey administered to higher education students across Tunisia. The survey was hosted on Google Forms, and participants were recruited through official university email lists, institutional social media pages, and on-campus flyers distributed with permission from student affairs offices at each participating institution. The platform’s built-in response-limiting settings restricted submissions to one response per device/browser session; no email address, phone number, or identification number was collected for deduplication purposes, consistent with the study’s data minimization approach. Participants completed four validated instruments and a brief sociodemographic section in a single session requiring approximately 20–25 min. The cross-sectional design enabled estimation of structural pathways and indirect effects through bootstrapped mediation analysis; its inherent limitation regarding temporal ordering is addressed in the Section 4.5.

### 2.5. Measurements

#### 2.5.1. Perceived Stress (PSS-10)

Perceived stress was assessed using the Arabic version of the Perceived Stress Scale (PSS-10) [10]. The negative subscale (perceived helplessness; PH) captures appraisals of uncontrollability and overwhelm across six items rated on a five-point Likert scale (0 = never; 4 = very often). The positive subscale (perceived self-efficacy; PSE) captures confidence in managing demands across four items on the same scale. Subscale scores were computed following the factor grouping specified in the original validation [10]. Internal consistency was acceptable in the present sample (Cronbach’s alpha = 0.74–0.77 across subscales), consistent with prior Arabic-language applications.

#### 2.5.2. Insomnia Severity (ISI)

Sleep disturbance was measured using the Arabic version of the Insomnia Severity Index (ISI), a seven-item self-report instrument validated for use in research and clinical settings [25]. Items assess difficulty initiating and maintaining sleep, early morning awakening, sleep dissatisfaction, daytime functioning interference, and distress related to sleep difficulties over the past two weeks. Total scores range from 0 to 28, with higher values indicating greater insomnia severity. As reported in Table 1, the value of M = 2.02 (SD = 0.62) for insomnia reflects the mean item-level score (i.e., the 7-item total divided by 7), not the 0–28 summed total; the corresponding mean ISI sum score is approximately 14.1, consistent with the moderate insomnia severity range. Internal consistency was strong in the present sample (Cronbach’s alpha = 0.84), consistent with the original validation [25].

#### 2.5.3. Psychological Distress (DASS-21)

Psychological distress was assessed using the Arabic Depression Anxiety Stress Scales (DASS-21), a validated 21-item instrument covering depression, anxiety, and stress subscales [26]. Items are rated on a four-point scale (0 = did not apply to me at all; 3 = applied to me very much or most of the time). A composite distress score summing all 21 items served as the primary outcome, consistent with its use as a global negative affect index in prior cross-sectional research.

#### 2.5.4. Physical Activity (IPAQ)

Weekly physical activity was assessed using the Arabic-validated form of the International Physical Activity Questionnaire (IPAQ), a widely validated self-report instrument for adult populations across diverse cultural settings [27,28]. Helou et al. [28] validated the long-form IPAQ in an Arabic-speaking sample; the present study administered the short form, which is noted as a measurement caveat in the Section 4.5. Responses were converted to metabolic equivalent task (MET) minutes per week following the standard IPAQ scoring protocol [27], and participants were classified into low, moderate, or high activity categories. Given the pronounced right-skew typical of IPAQ continuous scores, the resulting MET-minutes/week variable was natural-log transformed before correlation and structural equation modeling analyses to improve distributional properties; reported physical activity values in Table 1 and throughout the Results reflect this transformed scale rather than raw MET-minutes/week. MET-minutes per week (log-transformed) served as the continuous physical activity variable in all structural analyses.

### 2.6. Statistical Analysis

All analyses were performed in Jamovi (version 2.3.21.0; The Jamovi Project, Sydney, Australia). Descriptive statistics and distributional properties were computed for all variables. Internal consistency was evaluated using Cronbach’s alpha, McDonald’s omega, and Guttman’s lambda 6 (see Supplementary Table S1 for complete reliability indices across all four instruments). Pearson correlation coefficients were computed for all continuous variable pairs. Structural equation modeling (SEM) was used to simultaneously estimate direct and indirect pathways linking perceived helplessness, perceived self-efficacy, insomnia, physical activity, and psychological distress. The structural model was specified at the level of the five observed composite scores, with regression paths estimated among all pairs except a direct path between insomnia and physical activity, which was omitted a priori because these two variables were conceptualized as parallel, rather than sequential, mediators; this single constraint yields the model’s one residual degree of freedom. Model estimation used the unweighted least squares (ULS) method, which provides stable parameter estimates under conditions of moderate non-normality and ordinal measurement without requiring multivariate normality assumptions [29,30]. Model fit was evaluated using the chi-square test (interpreted alongside the approximate fit indices, since with a single degree of freedom the chi-square/df ratio is uninformative), comparative fit index (CFI > 0.95), Tucker–Lewis index (TLI > 0.90), root mean square error of approximation (RMSEA < 0.08), and standardized root mean square residual (SRMR < 0.08). Indirect effects were estimated via bootstrapping with 5000 resamples; 95% bias-corrected confidence intervals not spanning zero were used to infer statistical significance.

## 3. Results

### 3.1. Descriptive Statistics and Correlations

The sample reported relatively low mean scores on perceived helplessness (PH; M = 1.49, SD = 0.65) and perceived self-efficacy (PSE; M = 1.74, SD = 0.63). Psychological distress averaged 1.71 (SD = 0.55), and insomnia mean item scores were moderately elevated (M = 2.02, SD = 0.62 on the 0–4 item-level metric; equivalent total ISI score M = 14.14, SD = 4.34 on the conventional 0–28 scale). Physical activity (log-transformed MET-minutes/week; see Section 2.5.4) showed the greatest relative variability among the transformed variables (M = 7.84 log-MET-min/wk, SD = 1.12); the corresponding untransformed mean was approximately 2540 MET-min/week, consistent with typical IPAQ short-form values in young adult samples. Distributional analysis indicated near-normality for most variables, with slight negative skewness for PSE (−0.63) and mild positive skewness for psychological distress (0.77). Table 1 presents complete descriptive statistics; Supplementary Table S1 reports internal consistency indices (Cronbach’s alpha, McDonald’s omega, and Guttman’s lambda-6) for all instruments.

Pearson correlation analysis revealed marked differences between the two stress dimensions. PH showed strong positive associations with insomnia (r = 0.53, *p* < 0.01) and psychological distress (r = 0.52, *p* < 0.01), and a meaningful negative association with physical activity (r = −0.34, *p* < 0.01). PSE was positively but more weakly correlated with insomnia (r = 0.22, *p* < 0.01) and psychological distress (r = 0.25, *p* < 0.01), and negatively correlated with physical activity (r = −0.23, *p* < 0.01). This positive association between PSE and both insomnia and distress runs counter to PSE’s conventional protective framing in the stress literature and is interpreted with caution rather than presented as evidence of a protective effect; candidate explanations are considered in Section 4.2. The strongest bivariate association in the entire matrix was between insomnia and psychological distress (r = 0.58, *p* < 0.01). PH and PSE shared a modest positive correlation (r = 0.26, *p* < 0.01), confirming their empirical distinctiveness while acknowledging partial overlap. Table 2 presents the full correlation matrix.

### 3.2. Structural Equation Modeling

The hypothesized SEM demonstrated acceptable-to-good overall model fit: chi-square (1) = 10.82, *p* = 0.001; CFI = 0.994; TLI = 0.981; SRMR = 0.018; RMSEA = 0.076.The significant chi-square statistic reflects the large sample size rather than substantive model-data discrepancy; CFI and SRMR values meet criteria for excellent fit, while RMSEA (0.076) falls below the conventional 0.08 threshold. Because the model has a single degree of freedom, the RMSEA point estimate is interpreted with caution, as RMSEA is known to be unstable and upwardly biased at low degrees of freedom. The model is fully saturated at the level of the structural paths among the five composite scores, with one constraint, the omission of a direct insomnia–physical activity path (these were modeled as parallel rather than sequential mediators; see Section 2.6), producing the single residual degree of freedom.

Perceived helplessness produced a significant direct effect on psychological distress (beta = 0.25, *p* < 0.001) and substantial indirect effects: beta = 0.21 (*p* < 0.001) through insomnia and beta = 0.03 (*p* < 0.001) through physical activity. PH strongly predicted insomnia (beta = 0.51, *p* < 0.001), which in turn predicted psychological distress (beta = 0.40, *p* < 0.001). PH also significantly reduced physical activity participation (beta = −0.30, *p* < 0.001), and physical activity was inversely associated with distress (beta = −0.11, *p* < 0.001), a statistically reliable but small effect by conventional standardized-effect benchmarks, a point we return to in the Discussion.

**Figure 1:**
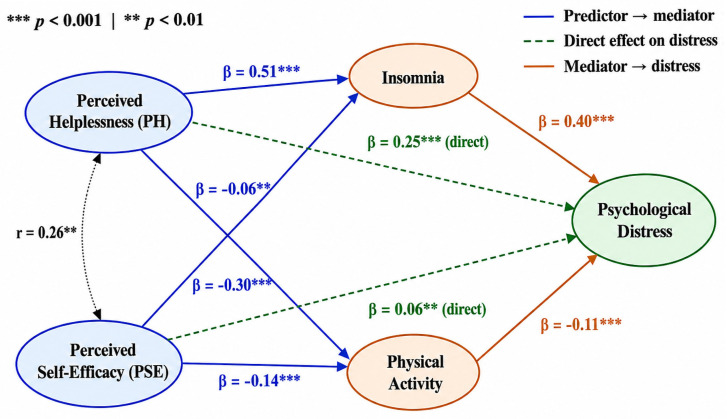
Structural equation model (SEM) depicting the relationships between stress, insomnia, physical activity, and psychological distress.

Perceived self-efficacy showed a statistically significant but substantially weaker direct effect on psychological distress (beta = 0.06, *p* = 0.002) and smaller indirect effects through insomnia (beta = 0.02, *p* = 0.006) and physical activity (beta = 0.01, *p* < 0.001). PSE produced only a modest positive association with insomnia (beta = 0.06, *p* = 0.006) and a weaker negative association with physical activity (beta = −0.14, *p* < 0.001). Across all pathways, PH exerted consistently larger effects than PSE, and insomnia was the dominant mediator throughout. The complete structural path coefficients and model fit indices are illustrated in Figure 1. Additionally, Table 3 presents all standardized path coefficients and indirect effect estimates with 95% bias-corrected confidence intervals, including total effects for each predictor.

## 4. Discussion

The present study provides a differentiated structural account of how two components of perceived stress translate into psychological distress through insomnia and physical activity in 1688 Tunisian young adults. Perceived helplessness emerged as the substantially more influential predictor, producing a strong direct effect and large indirect effects mediated principally by insomnia. Perceived self-efficacy contributed to the same outcomes but with considerably smaller effect sizes. Of the three directional hypotheses, H1 and H3 were supported, while H2 was not. H1 (PH exerts its primary indirect effect through insomnia) was confirmed with a large indirect effect (beta = 0.21), far exceeding PH’s indirect effect through physical activity (beta = 0.03). H2 (PSE exerts its primary indirect effect through physical activity) was not supported: Table 3 shows that PSE’s indirect effect through insomnia (beta = 0.02) was, if anything, marginally larger than its indirect effect through physical activity (beta = 0.01), the opposite of the hypothesized pattern. H3 (the insomnia pathway is substantially larger for PH than for PSE) was confirmed, both in absolute terms (PH’s insomnia-mediated indirect effect of 0.21 versus PSE’s 0.02) and in relative terms (insomnia is also PH’s dominant mediator by a wide margin over its own physical-activity pathway, 0.21 vs. 0.03, whereas PSE’s two indirect pathways are of comparable, both very small, magnitude). We revisit the implications of the unsupported H2 in Section 4.2.

### 4.1. Perceived Helplessness as the Predictor Most Strongly Associated with Psychological Distress

Perceived helplessness produced the strongest direct effect on psychological distress (beta = 0.25, *p* < 0.001) and the largest indirect effect through insomnia (beta = 0.21, *p* < 0.001) of any predictor in the model. By conventional thresholds, the total effect of PH on distress constitutes a medium-to-large association, consistent with theoretical predictions from Lazarus and Folkman’s [7] appraisal model, which positions the perception of resource inadequacy as a central contributor to sustained psychological strain. In a large cross-sectional study of Tunisian university students, Jelleli and colleagues [31] documented strong structural associations between problematic internet use, physical activity, and negative emotional states (depression, anxiety, and stress) that are broadly consistent with the centrality of behavioral and psychological regulatory processes observed in the present findings, though that study’s focus on problematic internet use differs from the stress-appraisal framework examined here. Chen and colleagues [11], in a 260-participant sample of Singaporean undergraduates, found that a stress-is-a-threat mindset mediated the relationship between stressful experience and psychological distress, while coping flexibility operated through a separate, challenge-mindset-linked pathway associated with lower distress; this dual-pathway structure offers a broadly compatible parallel to the present finding that helplessness (a threat-consistent appraisal) and self-efficacy (a coping-resource-consistent appraisal) show divergent associations with distress, reinforcing the value of treating appraisal and coping-resource constructs as distinct rather than collapsing them into a single score. Van Daalen and colleagues [32] extended this pattern to working adults, showing that perceived overload produced direct psychological exhaustion independently of available coping resources, a pattern consistent with the perceived helplessness direct effect observed here. Studies reporting smaller stress-distress associations typically collapse both PSS-10 subscales into a total score, attenuating the estimated direct effect by including the opposing self-efficacy component in the predictor. When helplessness is isolated, as in the present model, its association with distress is comparatively strong; given the cross-sectional design, this association should not be read as evidence of causal primacy. These findings suggest that interventions prioritizing stress-management skill development, including structured psychoeducation, regulation-focused cognitive training, and problem-solving protocols, may be a promising avenue for reducing psychological distress more efficiently than approaches focused solely on expanding coping resources, a hypothesis that would benefit from prospective evaluation.

### 4.2. Insomnia as the Key Mediating Pathway, and the Counterintuitive Role of Perceived Self-Efficacy

Insomnia emerged as the primary pathway through which perceived helplessness was associated with psychological distress (beta = 0.21, *p* < 0.001), and it produced the strongest bivariate association with distress of any variable pair in the model (r = 0.58, *p* < 0.01). This finding is broadly compatible with Van Someren’s [14] neurobiological framework, which positions insomnia not merely as a symptom of stress but as a disruption of the overnight emotional regulatory machinery, specifically the cortical restoration occurring during slow-wave sleep and the emotional memory consolidation occurring during REM sleep. As no physiological or longitudinal measures were collected in the present study, this mechanism should be understood as a plausible theoretical explanation drawn from the cited literature rather than a pathway confirmed by the present data. When stress-driven hypothalamic–pituitary–adrenal axis activation prevents adequate sleep, the brain’s capacity for next-day emotional regulation is theorized to be impaired, which may intensify negative affect and sensitive subsequent stress responses. Manzar and colleagues [15] documented a nearly identical indirect pathway in a university student sample, finding that insomnia mediated the stress-anxiety relationship with a statistically robust and clinically meaningful magnitude. Vandekerckhove and Wang [33] provided mechanistic grounding for this cycle, demonstrating that impaired REM sleep systematically degrades emotional regulation capacity and heightens reactivity to subsequent stressors. Arias-Carrion [34] extended this framework by identifying shared neuroinflammatory and HPA-axis hyperactivation pathways linking chronic insomnia to clinical depression and anxiety, offering a plausible account of the bidirectional, self-reinforcing character that has been proposed for the insomnia-distress relationship. The positive association between PSE and both insomnia (beta = 0.06) and psychological distress (beta = 0.06 direct; r = 0.25 and r = 0.22 bivariate) runs counter to PSE’s conventional framing as a protective factor and warrants explicit attention rather than passing mention. This pattern does not straightforwardly support the interpretation that higher self-efficacy is protective against sleep disruption in this sample, and we consider three non-exclusive candidate explanations. First, a suppression effect is plausible: because PSE correlates positively with PH (r = 0.26), some of PSE’s positive association with insomnia and distress may reflect shared variance with PH that produces a positive residual relationship once both predictors are modeled simultaneously, rather than reflecting a true positive effect of self-efficacy itself. Second, the cross-sectional design cannot rule out reverse causality, for instance, individuals already experiencing sleep disruption or distress over-reporting confidence items as a coping narrative or compensatory self-presentation. Third, a scaling or item-wording artifact in the PSS-10 positive subscale within this Arabic-speaking sample cannot be excluded and would warrant dedicated psychometric follow-up (e.g., differential item functioning analysis). We present these as candidate explanations for future investigation rather than as confirmed mechanisms, and we caution readers against interpreting the present PSE findings as evidence of a protective buffering role.

The finding that PSE produced a substantially weaker indirect effect through insomnia (beta = 0.02 vs. 0.21) is itself informative: rather than indicating a protective shielding effect, it indicates that PSE’s overall contribution to the insomnia pathway is small relative to PH’s, consistent with PH being the dimension most consequential for sleep disruption in this sample. Notably, this same insomnia route is also PSE’s larger of its two indirect pathways (0.02 versus 0.01 through physical activity; see Section 4 introduction and Section 4.4), so PSE does not contribute to distress predominantly via a protective, activity-based mechanism in this sample. These findings suggest that cognitive behavioral therapy for insomnia (CBT-I) may be a worthwhile candidate for integration into mental health programs targeting young adults with high perceived helplessness; we discuss feasibility considerations for this recommendation in Section 4.6 (Practical Implications).

### 4.3. Physical Activity as a Modest but Reliable Protective Association

Physical activity functioned as a consistent but small-magnitude protective correlate across all analytical approaches. Its negative association with psychological distress (beta = −0.11, *p* < 0.001) appeared in both the SEM pathways and the bivariate correlation matrix (r = −0.25, *p* < 0.01), and its indirect mediation of the PH-distress pathway (beta = 0.03, *p* < 0.001), while smaller than insomnia’s contribution, was statistically reliable across 5000 bootstrap resamples, though we note these effect sizes are small by conventional standardized-effect benchmarks and should be interpreted accordingly. This pattern is broadly directionally consistent with the wider literature on physical activity as a protective correlate of depression and anxiety symptoms, with the strongest effects generally reported for aerobic activity at moderate-to-vigorous intensity. Szuhany and colleagues [17] specifically documented that physical activity buffers against depression following major life stressors, providing resilience that operates through neuroplasticity and affect regulation pathways. The finding that PH negatively predicted physical activity participation (beta = −0.30, *p* < 0.001) is consistent with Stults-Kolehmainen and Sinha’s [18] comprehensive review, which identified motivational depletion, fatigue, and time displacement as the primary mechanisms through which perceived stress reduces physical activity in adults. Dhahbi and colleagues [35] showed that even modest increments in moderate-intensity aerobic activity produced measurable improvements in affect and cognitive function in adults, underscoring the accessibility of this protective threshold. The pattern identified here is consistent with a potential self-reinforcing cycle, in which high perceived helplessness is associated with reduced activity engagement, removing a small but reliable protective correlate of distress; longitudinal data would be needed to confirm this cyclical, reinforcing structure [5,19]. Lifestyle disruptions linked to academic and economic pressures compound this cycle in young adult populations [1,36]. Physical activity promotion should be considered a complementary, low-cost component of student mental health programming alongside, rather than in place of, sleep- and stress-focused approaches; students identified as physically inactive alongside elevated PH may represent a subgroup worth prioritizing for behavioral activation intervention, pending confirmatory study.

### 4.4. Differential Pathways and the Empirical Case for Subscale Distinction

The contrast between the two stress dimensions is the most theoretically informative feature of the present results. Perceived helplessness activated a considerably stronger pathway through insomnia and a secondary pathway through physical activity, with both indirect effects substantially larger than those produced by perceived self-efficacy. This asymmetry is consistent with the possibility of fundamentally different regulatory processes: helplessness is a passive appraisal of insufficient resources that is theorized to trigger sustained threat arousal and impair sleep through HPA-axis hyperactivation, while self-efficacy is an active appraisal of coping capacity that in this sample showed a comparatively weak and partly counterintuitive relationship with both pathways (see Section 4.2). Studies that collapse both subscales into a total PSS-10 score cannot detect this asymmetry [8,9,20], and critical intervention implications are lost as a result. Montero-Marin and colleagues [37] made an analogous observation in burnout research, demonstrating that the type of coping strategy deployed mattered more than coping quantity: avoidant coping produced outcomes indistinguishable from no coping at all, while problem-focused coping produced divergent trajectories. Applied here, targeting coping quantity in individuals with high helplessness and poor sleep would likely be insufficient; they may benefit more from sleep-specific and stress-regulation-specific interventions than from generic wellness programming. The structural model presented here offers a preliminary empirical basis for hypothesis-driven, differentiated programming: brief helplessness-focused screening could plausibly help direct students toward sleep-prioritized versus activity-prioritized programs, a possibility we believe merits prospective evaluation before implementation (see Section 4.6).

### 4.5. Limitations

Several methodological constraints qualify the present findings. The cross-sectional design precludes causal inference: the temporal sequence of stress, sleep disruption, activity decline, and distress cannot be confirmed without longitudinal data with appropriate time lags between measurement occasions. Future studies employing experience-sampling or ecological momentary assessment designs would capture within-person dynamics, resolving ambiguity about directionality. Second, all measures relied on self-report instruments, introducing shared-method variance that may inflate associations relative to multi-method designs. The IPAQ short form is known to overestimate physical activity levels relative to accelerometer-based assessment; future work should supplement self-report with validated wearable monitoring. Relatedly, the Arabic IPAQ validation cited for the present sample [28] formally validates the long-form questionnaire; the short form administered here draws on the original cross-cultural short-form validation [27] in the absence of an identified Arabic-specific short-form validation, which should be treated as an additional measurement caveat alongside the known overestimation bias of the IPAQ short form. Third, the convenience sample drawn from Tunisian higher education institutions, concentrated across 6 public institutions in three regions, limits generalizability to non-student young adults, vocational trainees, employed young adults, students at private institutions, and students from regions of Tunisia not represented in the present sample, all of whom may face different stress profiles and behavioral repertoires. Relatedly, the psychiatric exclusion criterion was operationalized via a single self-report screening item rather than clinical verification, which carries a risk of misclassification, including both false exclusions and undetected true cases, that could not be assessed in the present data. Fourth, the present analyses did not stratify by sex. This represents a more substantial limitation than a brief mention conveys, given the well-documented sex differences in insomnia prevalence, stress reactivity, and physical activity participation; future studies should test sex as a moderator of all structural pathways, with adequate power for subgroup comparison. Fifth, the self-efficacy subscale captures general coping confidence rather than specific coping strategy types; distinguishing problem-focused from emotion-focused and avoidant coping would enable more targeted characterization of which specific coping processes protect against distress [37,38].

### 4.6. Future Research Directions

Building on the limitations above, we highlight several priorities for future work. First, longitudinal or experience-sampling designs are needed to establish the temporal ordering of perceived helplessness, insomnia, physical activity, and psychological distress, and to test whether the structural pathways identified here hold directionally over time. Second, sex-stratified or multi-group SEM analyses should examine whether the present pathways, particularly the insomnia mediation of perceived helplessness, operate uniformly across male and female participants, given well-documented sex differences in sleep and stress reactivity. Third, the counterintuitive positive association between perceived self-efficacy and both insomnia and distress warrants dedicated follow-up, including psychometric investigation of the PSS-10 positive subscale in Arabic-speaking samples and designs capable of distinguishing suppression effects from reverse causality. Fourth, future studies should incorporate multi-method assessment, including accelerometer-based physical activity monitoring and, where feasible, polysomnographic or actigraphic sleep measures, to reduce shared-method variance. Finally, replication in non-student young-adult populations and in private-institution and geographically broader samples would help establish the generalizability of the present findings beyond the public, convenience-sampled cohort studied here.

### 4.7. Practical Implications

With the above caveats in mind, the present findings offer several hypothesis-generating implications for student mental health practice. Brief screening for perceived helplessness, rather than total PSS-10 scores alone, may help identify students for whom sleep-focused intervention is likely to be most relevant, given the substantially larger insomnia-mediated pathway observed for this dimension relative to perceived self-efficacy. Recommending CBT-I for students with elevated perceived helplessness is reasonable in principle, but broad implementation should account for provider training requirements and accessibility constraints, which vary substantially across university counseling settings and should be assessed before scale-up. Physical activity promotion, while showing only small effect sizes in the present model, remains a low-cost, low-risk complement to sleep- and stress-focused programming and can reasonably be offered universally rather than reserved for a specific subgroup. We emphasize that these are practice-relevant hypotheses derived from cross-sectional associations rather than validated intervention protocols, and that prospective or intervention-based studies are needed before any stratified triage approach is adopted in practice.

## 5. Conclusions

This cross-sectional study examined how perceived helplessness and perceived self-efficacy, the two factorially distinct dimensions of the PSS-10, relate to psychological distress in 1688 Tunisian young adults aged 18–23 years, using insomnia and physical activity as concurrent mediators within a structural equation model. Perceived helplessness emerged as the predictor most strongly associated with psychological distress, producing strong direct effects and large indirect effects mediated primarily through insomnia, with a secondary pathway operating through reduced physical activity. Perceived self-efficacy contributed to distress through the same mediating mechanisms but with considerably smaller magnitudes, and in a direction that did not consistently match its conventional protective framing (see Section 4.2), indicating that general coping resources did not, in this sample, function as a straightforward buffer against the effects of stress regulation deficits. Insomnia occupies a central role in this framework, showing the strongest association with distress of any variable examined; theoretically, it may function not merely as a byproduct of stress but as a mechanism that sustains and intensifies psychological distress by impairing overnight emotional regulatory processes, though confirming this temporal and mechanistic claim requires longitudinal and physiological data beyond the scope of the present design. Physical activity operated as a consistent but small-magnitude protective correlate across all analyses, and the finding that high perceived helplessness directly reduced physical activity participation is consistent with a behavioral pathway through which elevated stress co-occurs with reduced engagement in a protective health behavior, a pattern that future longitudinal work could test for evidence of progressive erosion over time. For practice, these results offer a hypothesis-generating basis for a stratified approach: students presenting with elevated perceived helplessness may be reasonable candidates for prioritized sleep-focused intervention, including CBT-I where provider resources allow, alongside structured stress-regulation skill training; we note in Section 4.7 that provider training and accessibility constraints should be assessed before broad implementation. Physical activity promotion should be positioned as a universal complementary strategy for all students rather than a stand-alone mental health intervention. For research, the integration of both PSS-10 subscales and two behavioral mediators within a single structural model establishes a useful starting point for mechanism-based analyses that move the field beyond unidimensional stress operationalization. Future longitudinal and multi-method studies should confirm the temporal ordering of these pathways, examine sex-based moderation, and test differentiated intervention protocols informed by the stress-dimension framework presented here.

## Figures and Tables

**Table 1:** Descriptive statistics for all study variables (n = 1688). M = mean; SD = standard deviation; PH = perceived helplessness; PSE = perceived self-efficacy; MET-min/wk = metabolic equivalent task minutes per week (log-transformed).

Variable	M	SD	Skewness	Kurtosis
Perceived Helplessness (PH)	1.49	0.65	−0.05	0.55
Perceived Self-efficacy (PSE)	1.74	0.63	−0.63	0.11
Psychological Distress	1.71	0.55	0.77	0.09
Insomnia (ISI, mean item score, 0–4)	2.02	0.62	0.43	−0.22
Insomnia (ISI, total score, 0–28)	14.14	4.34	0.43	−0.22
Physical Activity (MET-min/week, log-transformed)	7.84	1.12	0.34	−0.38

**Table 2:** Pearson correlation matrix for all study variables (n = 1688). PH = perceived helplessness; PSE = perceived self-efficacy. ** *p* < 0.01.

Variable	1	2	3	4	5
1. Perceived Helplessness (PH)	-	0.26 **	0.53 **	0.52 **	−0.34 **
2. Perceived Self-efficacy (PSE)	0.26 **	-	0.22 **	0.25 **	−0.23 **
3. Insomnia	0.53 **	0.22 **	-	0.58 **	−0.25 **
4. Psychological Distress	0.52 **	0.25 **	0.58 **	-	−0.25 **
5. Physical Activity	−0.34 **	−0.23 **	−0.25 **	−0.25 **	-

**Table 3:** Standardized path coefficients and bootstrapped indirect effects (5000 resamples). beta = standardized coefficient; SE = standard error; 95% CI = bias-corrected confidence intervals; PH = perceived helplessness; PSE = perceived self-efficacy.

Pathway	beta	SE	*p*	95% CI
**Direct Effects**				
PH -> Psychological Distress	0.25	0.03	<0.001	**[0.19, 0.31]**
PSE -> Psychological Distress	0.06	0.02	0.002	[0.02, 0.10]
PH -> Insomnia	0.51	0.03	<0.001	[0.45, 0.57]
PSE -> Insomnia	0.06	0.02	0.006	[0.02, 0.10]
PH -> Physical Activity	−0.30	0.03	<0.001	[−0.36, −0.24]
PSE -> Physical Activity	−0.14	0.03	<0.001	[−0.20, −0.08]
Insomnia -> Psychological Distress	0.40	0.03	<0.001	[0.34, 0.46]
Physical Activity -> Psych. Distress	−0.11	0.02	<0.001	[−0.15, −0.07]
**Indirect Effects (bootstrapped, 5000 resamples)**				
PH -> Insomnia -> Distress	0.21	0.02	<0.001	[0.17, 0.25]
PH -> Physical Activity -> Distress	0.03	0.01	<0.001	[0.01, 0.05]
PSE -> Insomnia -> Distress	0.02	0.01	0.006	[0.01, 0.04]
PSE -> Physical Activity -> Distress	0.01	0.00	<0.001	[0.00, 0.02]
**Total Effects (Direct + Indirect)**				
PH -> Psychological Distress (total)	0.49	0.03	<0.001	[0.43, 0.55]
PSE -> Psychological Distress (total)	0.09	0.02	<0.001	[0.05, 0.13]

## Data Availability

The data supporting the findings of this study are available upon reasonable request from the corresponding authors due to ethical and privacy restrictions related to participant confidentiality.

## Supplementary Materials

The following supporting information can be downloaded at: https://www.mdpi.com/article/10.3390/healthcare14142056/s1, Table S1: Internal consistency indices for all study instruments (n = 1688).
