# Supplementary material for: Distinct Stress Appraisal Dimensions, Insomnia, and Physical Activity as Differential Pathways to Psychological Distress: A Cross-Sectional Study in Tunisian University Students
Source: Healthcare (Basel). 2026 Jul 9;14(14):2056. doi: 10.3390/healthcare14142056 (PMC13409784; doi:10.3390/healthcare14142056)
Supplement: Supplementary file 1 [file healthcare-14-02056-s001.zip › healthcare-4354158-supplementary.pdf]

Supplementary Material

Table S1. Internal consistency indices for all study instruments (n = 1,688).

**Instrument / Subscale**	**α**	**ω**	**$\lambda6$**
PSS-10 – Perceived Helplessness (PH)	.77	.78	.77
PSS-10 – Perceived Self-efficacy (PSE)	.74	.75	.73
Insomnia Severity Index (ISI)	.84	.85	.84
DASS-21 (Total Psychological Distress)	.91	.92	.91
IPAQ (short form, log-transformed MET-min/wk)	—	—	—

α = Cronbach's alpha; ω = McDonald's omega; $\lambda6$ = Guttman's lambda-6. PH = perceived helplessness; PSE = perceived self-efficacy
